# Conserved OprF as a Selective Immunogen against *Pseudomonas aeruginosa*

**Published:** 2017-04-01

**Authors:** Ehsan Kazemi Moghaddam, Parviz Owlia, Abolfazl Jahangiri, Iraj Rasooli, Mohammad Reza Rahbar, Marjan Aghajani

**Affiliations:** 1 *Dept. of Microbiology, Faculty of Medicine, Shahed University, Tehran, Iran.*; 2 *Molecular Microbiology Research Center, Faculty of Medicine, Shahed University, Tehran, Iran.*; 3 *Applied Microbiology Research Center, Baqiyatallah University of Medical Sciences, Tehran, Iran.*; 4 *Dept. of Biology, Faculty of Basic Sciences, Shahed University, Tehran, Iran.*; 5 *Pharmaceutical Sciences Research Center, Faculty of Pharmacy, Shiraz University of Medical Sciences, Shiraz, Iran.*; 6 *Dept. of Physiology, School of Medicine, Tehran University of Medical Sciences, Tehran, Iran.*

**Keywords:** OprF Protein, *Pseudomonas aeruginosa*, Vaccine

## Abstract

**Background & Objectives:**

Due to the importance of *Pseudomonas aeruginosa* in severe inpatient infections and high mortality, the need for an efficient vaccine against these bacteria is increasing. In this regard, the general outer membrane porin of the most problematic microorganism *P. aeruginosa*, outer membrane protein F (OprF), is a good vaccine candidate.

**Methods:**

The databank of NCBI was used to retrieve protein sequences recorded for OprF in *P. aeruginosa.*The current study aimed at investigating the conservation of the OprF in 150 reference sequences, clinical, and environmental strains of *P. aeruginosa* from different countries via bioinformatic tools.T-COFFEE and PRALINE software were used for alignment.

**Results:**

Of these, 134 strains were isolated from clinical specimens and other strains from environmental samples. Evaluation of alignment by the mentioned software clearly showed that this protein was conserved. Antigenicity and grand average of hydropathicity were favorable.

**Conclusion:**

Conservation of OprF in all pathogenic and environmental strains of *P. aeruginosa* indicated that it can be considered as a good immunogen; however, the protectivity of OprF should be validated experimentally.

## Introduction

*Pseudomonas aeruginosa* is an important opportunistic pathogen. The bacteria can cause many problems in health care facilities, especially nosocomial infections. In recent years, *P. aeruginosa* showed multidrug resistance against routine antibiotics (1-4). Due to the role of the resistance and its ability in environmental adaptation, *P. aeruginosa* is widely distributed in hospitals (5). This problem is very acute in burn wards of hospitals (1-4). The prevention methods can be the best solution against *P. aeruginosa* infections (6). In other studies, many antigens such as outer membrane proteins (Omps), lipopolysaccharide, toxins, pili, and flagella are considered as targets for vaccine design (7-8). In recent years, outer membrane protein F (OprF) is known as one of the main factors in the immunity against *P.* *aeruginosa* infections (7). OprF has a special role in *P. aeruginosa* and binds the peptidoglycan to outer membrane. Removing OprF causes reshuffling of the outer membrane structure and morphology of organism (9). Homology modeling of OprF in *P. aeruginosa* shows that this protein has conserved and hydrophilic areas located outside the β-barrel area (9-11). To the authors` best knowledge; there is no comparative study on the conservation of this protein in a broad spectrum that could encompass a vast majority of the strains reported so far. Today, advances in genome sequencing and the development of biological technologies, created new methods to determine the conservation of antigens via bioinformatics (12-15). The current study designed an in silico approach to compare OprF sequences of the bacterial strains in different geographical regions. Hence, it would be possible to predict the similarity of the amino acid sequences of this protein to those of the strains reported in Iran to design a vaccine against OprF.

## Material and methods

**Available sequences and search in data bases**

OprF was searched in protein databank of NCBI (www.ncbi.nlm.gov) to obtain all of its recorded sequences. This search was limited to *P. aeruginosa*, and the full-length sequences were selected among reference and clinical strains. All amino acid sequences of OprF were retrieved and saved in FASTA format.

**Alignments**

To perform a more precise analysis on sequence similarity, alignment was run on the selected sequences (from previous step) by PRALINE (16) at http://www.ibi.vu.nl/programs/praline. This server can align up to 500 sequences each constituent 5000 amino acid.

Results built by this server could be represented by four important characteristics of amino acids in the protein sequence:

- Amino acid conservation
- Secondary structure
- Hydrophobicity
- Residue colour scheme

Another software with high accuracy for sequence alignment (T-COFFEE) was also employed (17). 

**Primary analysis of sequences**

Some physicochemical properties of proteins play an important role in the antigenicity, epitope, and B-cells stimulation. One of these properties is the charge of protein; ie, acidity or alkalinity. Properties such as molecular weight, isoelectric pH (PI), amino acid composition of the proteins, the number of positively and negatively charged amino acids, half-life of proteins, and protein instability indices were calculated by Protparam at http://web.expasy.org/protparam/ OprF sequence using Acc. No. NP_250468.1 in *P. aeruginosa* PAO1 (13-15).

**Antigenicity prediction**

The protein antigenicity was examined by VaxiJen (18) at http://www.ddg-pharmfac.net/vaxijen/VaxiJen/VaxiJen.html. Results above the threshold of 0.4 were proposed as antigen. This server can use the physicochemical properties to determine the antigenicity of a protein.

## Results

The current study investigated the extent of OprF conservation in clinical and environmental *P. aeruginosa *isolates*.* The amino acid sequences of OprF in 150 strains were studied:

Reference strains included 10 strains listed in Table 1. Reference sequences (RefSeq) represent genomic, transcription, and protein sequences of microorganisms that determine its bibliographic information and features. These reference sequences were collected at NCBI from the database and archived in International Nucleotide Sequence Database Collaboration (INSDC) (19).

**Table 1:** Information About the Reference Strains of *Pseudomonas aeruginosa*

Pseudomonas aeruginosa Strain	Accession Version Number	No.
**PAO1**	NP_250468.1	1
**PAO1-VE13**	YP_008705911.1	2
**PAO1-VE2**	YP_008693630.1	3
**PAO581**	YP_008557657.1	4
**c7447m**	YP_008551991.1	5
**Pseudomonas aeruginosa**	WP_034069203.1	6
**Pseudomonas aeruginosa**	WP_033973003.1	7
**Pseudomonas aeruginosa**	WP_034002471.1	8
**Pseudomonas aeruginosa**	WP_039843162.1	9
**Pseudomonas aeruginosa**	WP_003087843.1	10

*Clinical strains* included as follows:

A. Twenty-two pathogenic samples reported from Europe: Germany (five), England (ten), Italy (one), Netherlands (two), Spain (one), France (two), and Portugal (one)

B. Ninety-eight clinical samples reported from North America

C. Fourteen clinical samples reported from Asia: Japan (six), China (three), India (four), and Australia (one)

*Environmental strains* included 6 strains.

It should be noted that all of these strains were distinct and selection of any strain was according to the geographical area. Information on the clinical isolates of *Pseudomonas aeruginosa* is shown in Table 2.

**Table 2:** Information About the Distribution of Clinical Isolates of *Pseudomonas aeruginosa*

Year of Submission	Source	Accession Version Number	Country
**2012-2014**	(wound) (wound) (clinical *) (genitourinary system) (CF)	(CDM52442.1) (CDM46457.1) (CEI19826.1) (CDH71082.1) (AHC77743.1)	**Germany**
**2008-2014**	(outbreak in UK hospitals) (Ulcerative keratitis) (Cystic Fibrosis) (Clinical isolated) (Liverpool epidemic strain)	(CDI89967.1) (EFQ41449.1) (CAW28279.1) (KHE34418.1) (AHL14143.1, AHL08189.1, AHL02268.1, AHK96305.1, AHK90325.1, AHK84424.1)	**England**
**2006-2014**	(*) (Burn patient) (cystic fibrosis) (sputum) (wound) (blood) (*) (urine) (abscess) (ear) (genital) (ball) (nail bed) (urinary tract infection) (G-tube site) (corneal scraping) (vitreous fluid) (eye) (corneal ocular infection) (lung biopsy)	)AGN94862.1( (ABR82391.1, EKA53543.1, KFL13450.1, EHS42338.1, EHS36854.1, KJC19681.1, KJC16904.1, AJD64688.1, ETU89821.1, KAJ89582.1) (ABJ10962.1) (EJY60673.1, KEA25672.1, AJF49242.1, EIE44464.1, KEA24361.1, KEA10491.1, KEA09778.1, ERZ28007.1, ERZ36722.1, ERZ24153.1, ERX50757.1) (EYU07884.1, EZN80892.1, EZN74134.1, ETV39405.1, ETV07925.1, ERW32618.1) (KKJ52823.1, KKJ46459.1, ETV35921.1, ETV35355.1, ETV16230.1, ERY97934.1,) (KEI26513.1, ERX22014.1) (EZP22391.1, EZP12828.1, EZP14721.1, EZO99483.1, EZO95118.1, EZO86436.1, EZO82517.1, EZO75693.1, EZO69652.1, EZO67036.1, EZO57980.1, EZO48396.1, EZO53197.1, EZO51069.1, EZO37978.1, EZO37459.1, EZO21029.1, EZO16533.1, EZO08668.1, EZO13890.1, EZO04400.1, EZN89150.1, EZN95416.1) (EZN75858.1, EZN55327.1, ETV47231.1, ETV14786.1, ETU85059.1, ERY79601.1) (EZN65786.1, ERZ08353.1, ERY97607.1) (EZN54663.1, ERY80772.1) (EZN48086.1) (ETV62667.1, ETV05174.1) (ETV53437.1) (ERZ21361.1, ERZ11526.1, ERX35118.1, ERX55571.1, ERX61779.1) (ERY96687.1) (ERY66449.1, ERY52232.1, ERY45831.1, ERY15660.1, ERY25231.1) (ERY63790.1) (ERY47099.1, ERY32203.1, ERY30400.1, ERY11829.1, ERY06602.1 ERY04098.1, ERX94226.1) (ERX39027.1, ERX35485.1) (ERW07241.1)	**United States of America**
**2011-2015**	(*) (Nosocomial pathogen worldwide)	)GAA17329.1, BAQ40580.1, BAK89536.1, BAR68460.1( (BAP51451.1,BAP23858.1)	**Japan**
**2014**	(*)	AHW71751.1	**Canada**
**2012-2013**	(*) (Septicemia patient)	(AGI82172.1, AHA27099.1) (EOQ79915.1)	**China**
**2008**	Blood from a hospitalized oncology patient	ENH94677.1	**Italy**
**2013**	Cystic fibrosis	CCQ83615.1	**Australia**
**2015**	(*)	AKE70517.1, AKF99939.1	**Netherland**
**2014**	Blood culture of Spanish hospitals	CEI76311.1	**Spain**
**2014**	(*) (Blood sample from patient septicaemia)	(KHE59287.1, KHE56247.1, AID84511.1) (KFF34883.1)	**India**
**2013**	(nose of a patient in the surgical intensive care unit) (Asymptomatic carrier)	(ESZ83328.1) (ESR98694.1)	**France**
**2011**	Patient whit pneumonia	ESQ63932.1	**Portugal**

* Unknown source of the clinical sample

These results show that the samples were recorded from 2006 to 2014; and most of them were related to the USA. 

**Fig 1:**
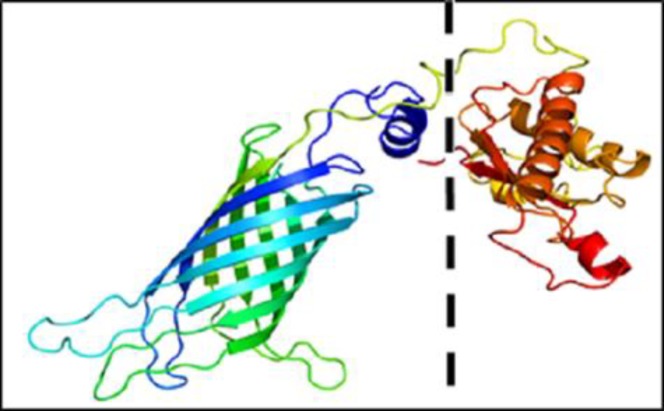
3D structure of OprF

Information about the environmental isolates of *Pseudomonas aeruginosa *is shown in Table 3. The alignment results revealed that the amino acid sequences of the OprF were fully conserved in all of the 150 strains (data not shown). Analysis of physicochemical properties of OprF by ProtParam software showed that the sequence of mature OprF possessed 326 amino acids (Fig. 1) with a molecular weight of 35250.6 Dalton and an isoelectric pH (PI) of 4.86. The total number of negatively (Asp + Glu) and positively charged amino acids (Arg + Lys) in the protein were calculated as 47 and 32, respectively. Its molecular formula and total atoms were C_1528_H_2356_N_436_O_508_S_9_ and 4837, respectively. The instability index (II) of protein was 28.13. Proteins with instability index smaller than 40 were predicted as stable. Grand average of hydropathicity (GRAVY) was -0.443. The antigenic probability of OprF was estimated 0.8010 by VaxiJen. 

**Table 3:** Information about the Environmental Strains of *Pseudomonas aeruginosa*

Year of Submission	Source	Accession Version Number	Country
**2012-2014**	(Water isolate)(air isolate)(soil isolate)(environmental isolate)	(CDH77900.1) (EKA49649.1) (EKA32488.1) (ETU77664.1, ETU74634.1)	United States of America
**2013**	Water	AID72990.1	Germany

## Discussion

OprF is one of the main outer membrane proteins in *P. aeruginosa* that plays an important role in quorum sensing and biofilm formation. Moreover, the porin function is observed in facilitated diffusion. The most important role of this protein is to connect peptidoglycan to outer membrane, which is very important in cell morphology. Moreover, OprF binding to gamma interferon causes infection distribution (5). The physicochemical analysis showed that OprF is a stable protein. The instability index provides an estimate of the stability of the protein in a test tube. Statistical analysis of 12 unstable and 32 stable proteins revealed that there are certain dipeptides with significantly different occurrence in the unstable proteins compared with those of the stable ones (20).

 It is shown that binding of the antibody to the protein causes deformation of bacteria and it is the loss of biological actions and also block binding of interferon-gamma to the cell (5, 8). The GRAVY value for a peptide or protein is the sum of hydropathy values of all the amino acids (21). 

The higher negative number of GRAVY of the epitope is more suitable for reacting with antibody. Also, antigen probability estimated by VaxiJen showed that this protein was highly antigenic. Consequently, the protein was considered as a good candidate for vaccine, and production of such a vaccine is in preclinical phase (22). After years of study on the lipopolysaccharide (LPS) vaccine adjuvant, the use of such vaccines failed due to existence of different serotypes of O-antigens (4). Therefore, the main question of the current study was that whether or not such a problem would happen to OprF vaccine. Sequence similarity search was done to reach a conserved region in the sequence. This area should be conserved in all or a vast majority of pathogenic strains.

 The results of the current study revealed that the protein sequences of OprF in 134 clinical strains were highly conserved. Recently, the analysis of the anti-P. aeruginosa systemic and lung mucosal immunity elicited by a non-human primate-based Ad serotype C7 vector expressing OprF (AdC7OprF) of *P. aeruginosa* showed that direct administration of AdC7OprF to the respiratory tract resulted in an increase of OprF-specific IgG in serum, OprF-specific IgG and IgA in lung epithelial lining fluid (ELF), and OprF-specific INF-γ in lung T-cells compared to immunization with Ad5OprF, and survival following the challenge with a lethal dose of *P. aeruginosa* (23). In a study by Worgall et al. (24), employment of a 14-amino acid epitope of *P. aeruginosa *OprF (Epi8) resulted in boosting of the anti-OprF humoral and anti-Epi8 cellular responses.

 Recombinant OprF/I vaccine inhibit *P. aeruginosa* binding to IFN-gamma, suggesting a mechanism by which the OprF/I vaccine confers protection against *P. aeruginosa* infection (9). 

This evidence suggested that employment of an OprF vaccine may be effective in prevention of *P. aeruginosa *infections*, *worldwide*.*
